# Theoretical Analysis and Numerical Simulation of the Motion of RDX Deflagration-Driven Flyer Plate Based on Laser-Initiated Micro-Pyrotechnic Devices

**DOI:** 10.3390/mi14050917

**Published:** 2023-04-24

**Authors:** Mingchun Xian, Kang Zhao, Xuwen Liu, Yangang Meng, Junyao Xie, Jingwei Li, Lele Tong, Meng Huang, Lizhi Wu

**Affiliations:** 1 School of Chemistry and Chemical Engineering, Nanjing University of Science and Technology, Nanjing 210094, China; 2 Sichuan Aerospace Chuannan Pyrotechnics Co., Ltd., Luzhou 646000, China; 3 State Key Laboratory of Precision Blasting, Jianghan University, Wuhan 430056, China

**Keywords:** laser-initiated, deflagration-driven, flyer plate, powder burn

## Abstract

Miniaturized laser-initiated pyrotechnic devices have great application prospects in aerospace and modern weapon systems due to their excellent energy output performance and reliability. In order to develop a low-energy insensitive laser detonation technology based on a two-stage charge structure, it is important to deeply analyze the motion law of a titanium flyer plate driven by the deflagration of the first-stage charge (RDX). The effects of the charge mass of RDX, flyer plate mass, and barrel length on the motion law of flyer plates were studied through a numerical simulation method based on the Powder Burn deflagration model. The consistency between the numerical simulation and the experimental results was analyzed using the paired t confidence interval estimation method. The results show that the Powder Burn deflagration model can effectively describe the motion process of the RDX deflagration-driven flyer plate with a 90% confidence level, and its velocity error is ≤6.7%. The speed of the flyer plate is proportional to the mass of the RDX charge, inversely proportional to the mass of the flyer plate, and exponentially related to its moving distance. As the moving distance of the flyer plate increases, the RDX deflagration products and air in front of the flyer plate are compressed, which inhibits the motion of the flyer plate. In the optimum state (the mass of the RDX charge is 60 mg, the mass of the flyer is 85 mg, and the length of the barrel is 3 mm), the speed of the titanium flyer reaches 583 m/s, and the peak pressure of the RDX deflagration reaches 2182 MPa. This work will provide a theoretical basis for the refined design of a new generation of miniaturized high-performance laser-initiated pyrotechnic devices.

## 1. Introduction

Energetic materials can ignite or detonate when irradiated by laser light [1,2,3,4,5]. The miniaturized low-energy insensitive laser detonator designed based on this principle has the advantages of low-energy input and high-energy output, and can also ensure high safety and reliability of the igniter system [6,7,8]. Laser detonation has unparalleled advantages over conventional bridge wire detonation, such as characteristics of static-discharge proof and stray-current free, smaller size, reusability, easy-to-achieve multi-point synchronous ignition and in-line ignition, etc. [9,10,11]. In addition, it can provide technical support for research of the micro-scale detonation process and the development of new explosives. Therefore, laser-initiated micro-pyrotechnic devices have great application prospects in aerospace and weapon systems [11].

The first generation of laser detonators used primary explosives as the initial charge, so they could be initiated by low-power (less than 5 W) semiconductor lasers. They are compact in structure, low in power consumption, and have good engineering application value [12,13]. However, the defects of first-generation laser pyrotechnic products are also obvious, the most prominent of which is the safety issue. The sensitivity of primary explosives used in traditional laser detonators greatly limits their application scenarios. In order to solve this problem, researchers have conducted much research on how to realize laser pyrotechnics without a sensitive charge. Among these explorations, there are two most promising development directions: high-power laser detonation technology, which is characterized by relying on laser energy to directly drive flyers to detonate explosives, and low-energy insensitive laser detonation technology characterized by a two-stage charge structure [14]. The laser directly drives the flyer to detonate the explosive, which can directly realize the detonation of the highly insensitive explosive, however, this technology generally requires the power density of the laser to be greater than 10^6^ W/cm^2^. Therefore, the size of the laser will be large, which makes this technology difficult to achieve in engineering applications at present. The low-energy insensitive laser detonation technology based on the two-stage charge structure has a relatively low initiating power, which is comparable to the first generation of laser pyrotechnics, and has considerable application prospects, so it is a key direction of current research.

H. Moulard et al. [15] developed the technology of insensitive laser explosives based on the structure of a two-stage charge. A laser-initiated detonator based on driving the stepped flyer plate to strike and detonate the RDX by igniting HMX with a laser is proposed. Xian and Meng et al. [16] explored the shock initiation law of the insensitive laser micro-initiator based on the two-stage charge structure, studied the shock wave convergence effect when the flyer plate hit the second-stage charge, and discussed the initiation criteria. Bu et al. [17] conducted an experimental investigation into detonating the secondary charge by impacting the primary charge with a laser and then driving the flyer to detonate the secondary charge. Through the design of the structure of the prototype, the correlation between the size of the barrel and the flyer and the speed of the flyer is obtained. Wang [18] investigated the process where air pressure generated by the combustion of excitation powders drives the movement of the flyer, thereby colliding with the explosive and causing an explosion. The effects of the speed, diameter, and thickness of the flyer on the impact pressure and initiation performance were obtained through the test method.

In order to conduct in-depth research on the laser detonator based on the two-stage charge structure, in addition to the experimental investigation, scholars have also conducted related research on numerical simulations of the motion process of the flyer driven by explosive energy. Chen et al. [19] studied the process of a detonation-driven stainless steel flyer by numerical simulation method. Wang et al. [20] deduced the final velocity formula of the flyer directly driven by laser on the basis of the law of conservation of energy and a Gurney formula. Liu [21] used Ansys/LS-DYNA to simulate the motion of flyer plates of different materials driven by lead nitride explosions. Based on Lawrance modeling, Chen [22] established a numerical model for the movement of a laser-driven composite flyer plate. Jiang et al. [23] simulated the motion process of the flyer plate driven by PETN with different densities through Ansys/LS-DYNA.

However, the above-mentioned reports all assume that the motion law of explosive expansion is in a completely ideal detonation state, and use the JWL equation of state to describe the work law of the expansion of the explosive. Energy loss is closely related to the setting of boundaries. In traditional research schemes, the casing of the detonator is often simplified as a no-reflection boundary condition. These boundary conditions did not consider energy loss during the process. In this work, the setting of boundary conditions takes into account the process of the casing absorbing the energy of the explosive during the reaction (i.e., the process of energy loss of the explosive), which is more in line with the actual situation. The effect of actual energy losses on the growth of the detonation and the movement of the flyer plate has not been adequately considered. This work innovatively conducted a numerical simulation study of a model for the motion of a flyer plate driven by explosive deflagration. The motion law of the deflagration-driven flyer of the first-stage charge (RDX) in low-energy insensitive laser detonation technology based on a two-stage charge structure was deeply explored to guide the refined design of laser pyrotechnics. The motion process of the flyer plate driven by RDX deflagration was simulated using the Powder Burn model [24,25]. According to the effect of charge mass, flyer plate mass, and accelerating chamber length on flyer plate motion, samples with different gradients were designed for verification and model correction, providing theoretical support for research on laser detonators with low-energy consumption and insensitive charges.

## 2. Experimental Device and Numerical Modeling

### 2.1. Prototype and Experimental Method

#### 2.1.1. Prototype

**Figure 1:**
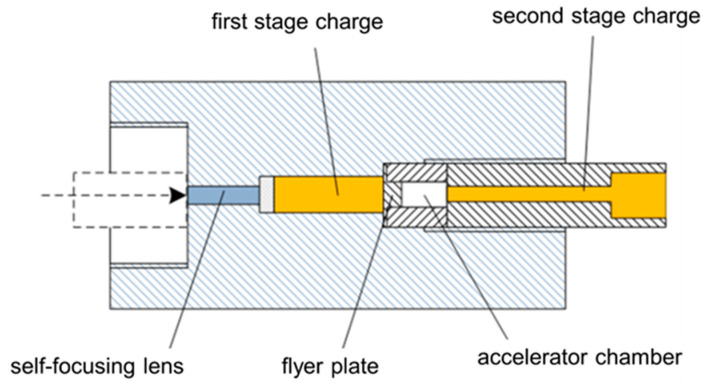
Schematic structure of a laser detonator.

The structure of the prototype of the low-energy insensitive laser detonator is shown in Figure 1. The prototype mainly includes a self-focusing lens, a first stage charge (B/KNO_3_+RDX), a flyer plate, an accelerating chamber, and a second stage charge (RDX/HMX). Its working principle is that the first-stage charge absorbs laser energy and deflagrates, and the huge pressure generated by the deflagration shears the flyer plate, causing it to hit the second-stage charge at high speed due to its acceleration through the barrel and detonate the second-stage charge.

**Figure 2:**
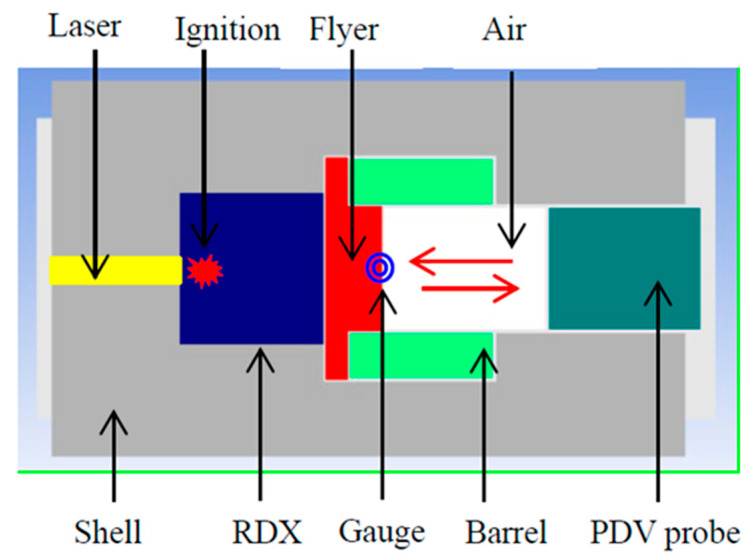
Schematic structure of the test prototype.

As shown in Figure 2, the structure of the test prototype is similar to that of the low-energy insensitive laser detonator prototype, of which the difference is that the second-stage charge of the detonator is canceled and replaced with a PDV (photonic Doppler velocimetry) probe for testing the velocity of the flyer plate.

#### 2.1.2. Test Device and Method

**Figure 3:**
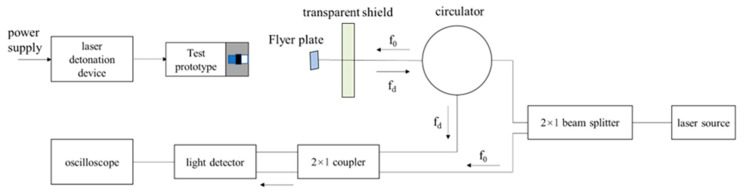
Test principle schematic diagram of PDV.

The test device consists of a PDV test system, a laser device, and a test prototype. The test prototype is detonated by laser, and the fiber optic probe of the PDV test system is fixed on the flight axis of the flyer plate and aligned with the exit of the barrel to obtain the speed data of the flyer plate in the whole process. The test device is fixed on the test bench through a fixed tool to reduce the interference of non-axial vibration during the test and facilitate the light positioning of the PDV test system. A schematic diagram of the PDV test process is shown in Figure 3.

### 2.2. Numerical Model

**Figure 4:**
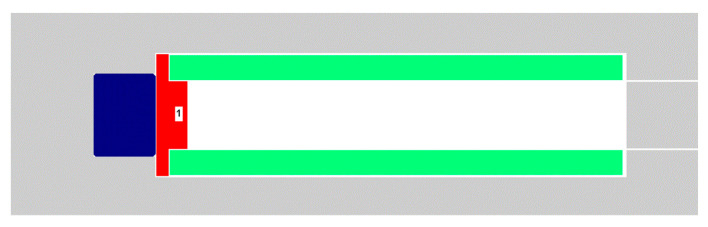
Schematic diagram of the simplified numerical simulation model.

The three-dimensional model can be simplified to a two-dimensional axisymmetric model. The ignition range is set to a 1 mm × 1 mm area near the laser emission position. Since the flyer plate, shell, and other components are clearance fit, explosive gas is released, so there is a gap of 0.1 mm between the structural parts. In order to study the variation law of velocity and kinetic energy in the accelerating chamber with the moving position of the flyer plate, the length of the accelerating chamber is set to 21 mm, and the numerical simulation observation point is located at the front center of the flying plate (corresponding to monitoring point 1). The simplified numerical simulation model is shown in Figure 4.

**Figure 5:**
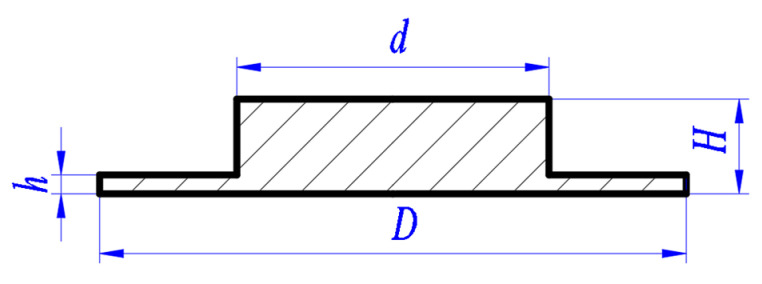
Schematic diagram of the flyer plate structure.

The charge is refined RDX of which the average particle size is less than 20 μm. RDX is pressed into the shell, the charge diameter ϕ is 2 mm, and the bulk density is 1.6 g/cm^3^. The charge mass is controlled by changing the charge length. The material of the flyer plate is titanium alloy, and the material of the barrel and the shell is stainless steel. The speed of the flyer plate decreases significantly with the increase of the length of the accelerating chamber, but the aperture of the accelerating chamber has little effect on the speed of the flying plate [20]. Therefore, the aperture of the accelerating bore is consistent with the minor diameter of the flyer plate. The schematic diagram of the flyer plate is shown in Figure 5, where *H* is the total thickness of the flyer plate, h is the shear thickness, *d* is the minor diameter, and *D* is the major diameter. Unless otherwise specified, the minor diameter *d* is 3.3 mm, the major diameter *D* is 6 mm, the total thickness *H* is 1.5 mm, and the shear thickness *h* is 0.6 mm.

**Figure 6:**
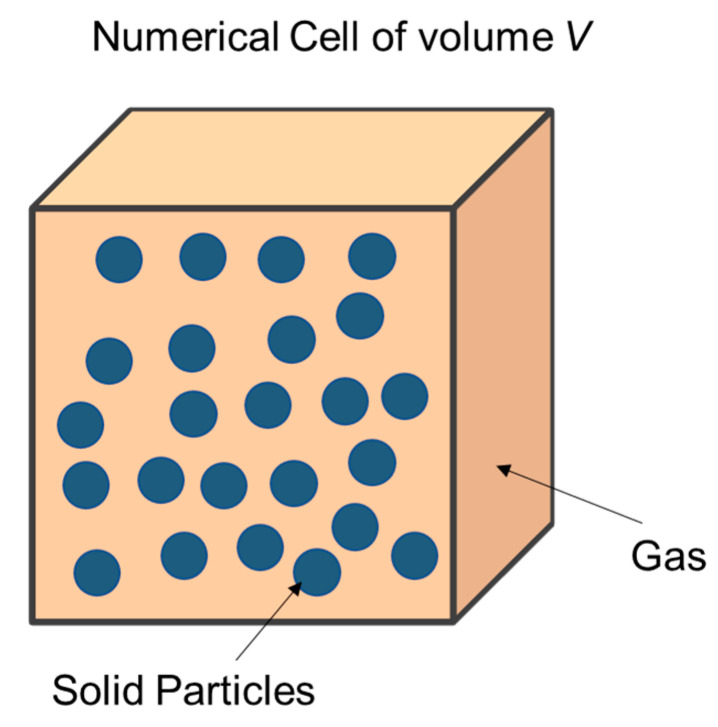
Schematic diagram of Powder Burn model unit in reaction.

The RDX deflagration state equation is described with the Powder Burn model. An arbitrary Lagrange–Euler (ALE) algorithm was adopted, and the grid size is 0.1 mm. The Powder Burn model is mainly used to simulate the macroscopic reaction process of the deflagration of energetic materials such as gun propellants and solid propellants, and there are both gas and solids in the unit [26]. The schematic diagram of the unit of the Powder Burn model in the reaction is shown in Figure 6.

Due to the fact that the loading density of unreacted RDX is actually controlled by the compaction pressure, in which the higher the pressure, the higher the loading density, this is a small deformation compression process, and the pressure is independent of the internal energy of the explosive. Therefore, in the Powder Burn model, the solid-state equation for unreacted RDX is the solid linear elastic state equation (Linear EOS), as shown in Formula (1), which is used to describe only the pressure of unreacted RDX related to the density. The JWL state equation is suitable for describing detonation processes with detonation pressure peaks in the range of tens of GPa. When the detonation pressure peak is less than 1 GPa or a few GPa, the gas state equation during the reaction is better described by the slow-burning process depicted by the exponential EOS, as shown in Formula (2).

(1) $$P=K\left(\frac{\rho }{\rho_{0}}-1\right)$$

where *K* is the bulk modulus of the material, $\rho_{0}$ is the initial density of the material, *P* is the pressure of the unreacted solid, and ρ is the density of the unreacted solid.

(2) $$P_{g}=\rho_{g}e_{g}Exp\left(\rho_{g}/D\right)$$

where *Pg*, $\rho_{g}$ and $e_{g}$ represent the pressure, density, and specific internal energy of the gas product after the RDX reaction, respectively, and *D* is a constant related to the reactant.

The burning rate (chemical reaction rate) is a segmented linear function, and the combustion rate mainly depends on the gas pressure *P_g_*, represented by data points:

(3) $$H\left(P_{g}\right),\left\{\left(P_{i},H_{i}\right)/1\leq i\leq 10,i\in N\right\}$$

where *P_i_* and *H_i_* represent the gas pressure and chemical reaction rate of the *i*-th data point on the *H*(*P_g_*) curve.

The reaction proportion change rate equation belongs to the macroscopic chemical reaction rate equation, which determines how much solid reactant is converted into gas product in each time step of the unit cell and can be expressed as

(4) $$F^{\prime }\left(t\right)=G{\left(1-\alpha F\left(t\right)\right)}^{c}H\left(P_{g}\right)$$

where G is the ignition growth parameter, c and α are constants related to the growth of reaction proportion. For spherical particles, G = 3⁄r, *c* = 2⁄3, and *α* = 1.

The ignition velocity is the speed of the combustion wave front and can be expressed as

(5) $$v\left(t\right)=C_{1}+C_{2}\left(H\left(P_{g}\right)\right)\left(1+\gamma \left(\rho_{s}\right)\right)$$

where *C*_1_ and *C*_2_ are constants related to the material, and *γ*(*ρ_s_*) is a custom equation, where *ρ_s_* is the average density of remaining solid in the unit cell.

Since the performance of RDX and HMX is similar; the main parameters of RDX in the Powder Burn model are appropriately modified on the basis of the parameters of HMX; as shown in Table 1. The remaining parameters of the Powder Burn model of RDX are consistent with those of HMX (95%)/HTPB (5%) in the reference [24].

The materials of the flyer plate, accelerating chamber, and shell are all metal, and are described by the Mie–Gruneisen equation of state, Johnson–Cook strength model, and erosion strain failure model. The ideal gas state equation is used to describe air, which is mainly used to simulate the convective diffusion and interaction among the structures.

The equation of state in Mie–Gruneisen form is as follows:

(6) $$p=\frac{\rho_{0}C_{0}^{2}\mu \left(1+\mu \right)}{{\left[1-\left(S-1\right)\mu \right]}^{2}}+\gamma \rho \left(e-\frac{1}{2}\frac{p_{H}}{\rho_{0}}\left(\frac{\mu }{1+\mu }\right)\right)$$

In the formula, *μ* = ρ⁄($\rho_{0}$−1), $\rho_{0}$ is the initial density of the material, *γ* is the Gruneisen coefficient, *C*_0_ is the intercept of the *u_s_* − *u_p_* curve, *S* is the coefficient of the slope of the *u_s_* − *u_p_* curve, *p* is the pressure, e is energy.

The yield stress of the Johnson–Cook model can be expressed as

(7) σY=(A+Bε¯pn)(1+Cln⁡ ε˙*)(1−THm)

In the formula, *A* is the initial yield stress of the material at the reference strain rate ${\dot{\varepsilon }}_{0}$ and the reference temperature $T_{H}$. *B* and *n* are the strain hardening modulus and hardening exponent of the material at the reference strain rate ${\dot{\varepsilon }}_{0}$ and the reference temperature $T_{H}$. $T_{H}$ is calculated through $T_{H}=\left(T-T_{room}\right)/\left(T_{melt}-T_{room}\right)$, while ${\dot{\varepsilon }}^{*}$ is the normalized effective plastic strain rate, calculated through ${\dot{\varepsilon }}^{*}={\bar{\dot{\varepsilon }}}^{p}/{\dot{\varepsilon }}_{0}$. *C* is the material strain rate strengthening parameter, ${\bar{\varepsilon }}_{p}^{n}$ is the effective plastic strain, and *m* is the thermal softening parameter of the material.

The main parameters of the Mie–Gruneisen equation of state are shown in Table 2. The main parameters of the Johnson–Cook strength model and failure model are shown in Table 3. Unspecified parameters are all defaulted to 0.

### 2.3. Paired t Confidence Interval Estimation Method

In order to analyze the validity of the numerical simulation results relative to the experimental results, when the numerical simulation data sequence is correlated with the experimental data sequence, the paired t confidence interval estimation method is adopted, which is applicable and reliable [29]. After the sample size is set to *n*_1_ = *n*_2_ = *n*, the numerical simulation data sequence $\left\{X_{1i}\right\}$ and the experimental data sequence $\left\{X_{2i}\right\}$ is paired to form a new relative error sequence $\left\{Y_{i}\right\}$:

(8) $$\left\{Y_{i}\right\}=\left\{X_{1i}\right\}-\left\{X_{2i}\right\}$$

At the significance level α, the confidence level is $\left(1-\alpha \right)$, and the confidence interval of the relative error sequence $\left\{Y_{i}\right\}$ is

(9) $$\bar{y}\left(n\right)\pm t_{\alpha /2}^{n-1}\bar{\frac{S^{2}\left(n\right)}{n}}$$

where $\bar{y}\left(n\right)$ is the sample mean of $\left\{Y_{i}\right\}$; $t_{\alpha /2}^{n-1}$ is the *α*/2 quantile of the *t* distribution with freedom degree of *n* − 1, and $S^{2}\left(n\right)$ is the sample variance of $\left\{Y_{i}\right\}$:

(10) $$S^{2}\left(n\right)=\frac{1}{n-1}\sum_{i=1}^{n}{\left(y_{i}-\bar{y}\right)}^{2}$$

## 3. Results and Discussion

### 3.1. The Process of RDX Deflagration Driving the Flyer Plate and the Validity Analysis of the Model

**Figure 7:**
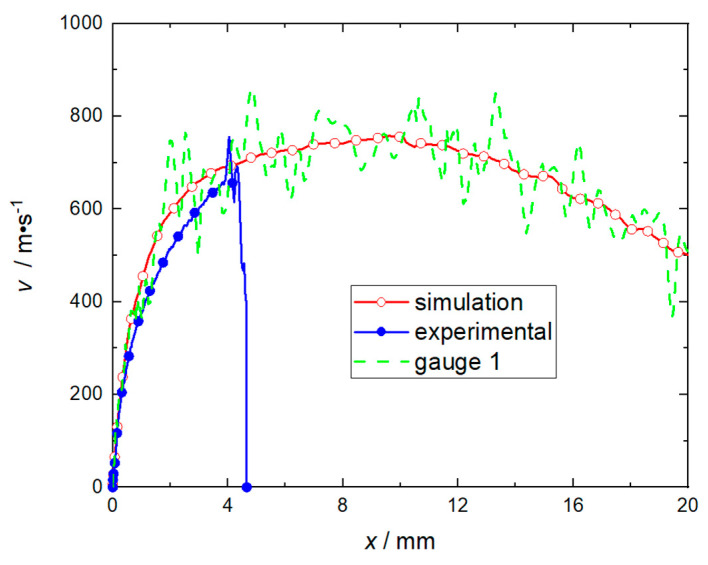
Velocity−distance curve of flyer plate.

Through numerical simulation, the velocity data of the flyer plate is calculated when the charge of RDX is 60 mg, the mass of the flyer plate is 85 mg (corresponding to a total thickness H of 1.5 mm), and the length of the accelerating chamber is 21 mm. The experimental data are the average values of 5 rounds, and the corresponding barrel length is 3 mm. The sample volumes n1, n2, n of the paired t confidence interval estimate are all 13, and the corresponding displacement gradient is 0.25 mm. The velocity-displacement curve of the flyer plate moving in the accelerated chamber is shown in Figure 7. When the significance level α=0.1, $t_{\alpha /2}^{n-1}$ is 1.3526. The confidence interval of $\left\{Y_{i}\right\}$ calculated by t confidence interval estimation is (−26.1~57.8) m/s, and the speed error when the flyer plate moves 3 mm is 2.1%. Since the confidence interval includes 0, it can be judged that there is no difference in performance between the two sets of data at a 90% confidence level. The acceptable range of experimental error is (−60~+60) m/s. Since the confidence interval meets the requirements of the acceptable accuracy range, it can be judged that the numerical model is valid, and the model’s reliability $P_{\alpha }$ is higher than 90%.

**Figure 8:**
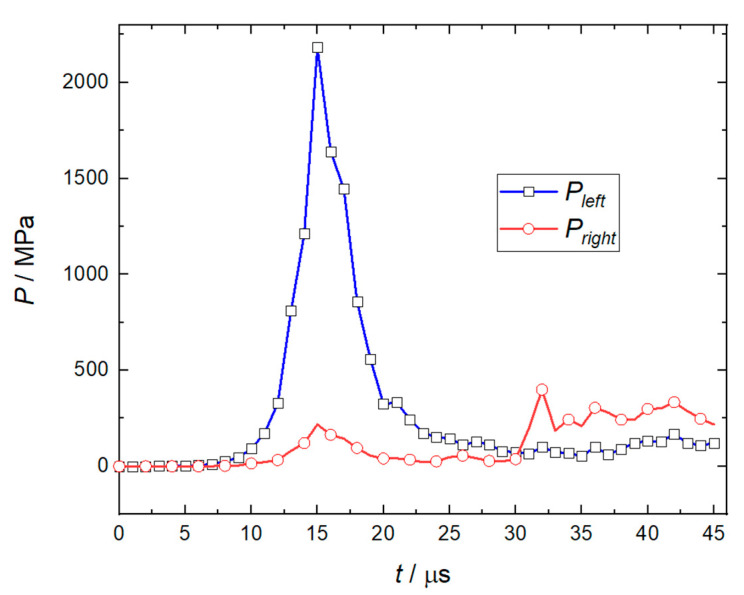
Peak deflagration pressure curve of RDX explosive.

**Figure 9:**
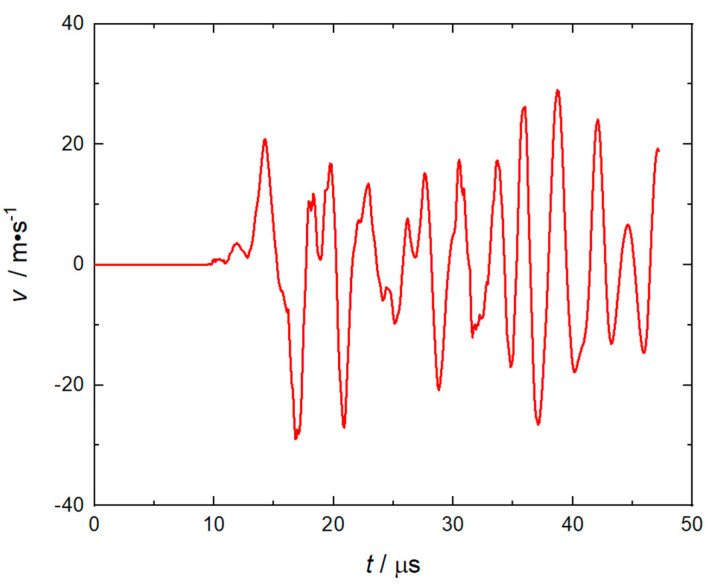
Velocity−distance curve in y direction of flyer plate.

The numerical simulation results shown in Figure 7 indicate that when the flyer plate is sheared, the velocity fluctuation at monitoring point 1 at the center of its front edge is apparent, as the pressure changes on the left and right sides of the flyer plate are relatively severe, especially when the flyer plate has been sheared, the peak value of the RDX deflagration pressure will reach the maximum, as shown in Figure 8. When the flyer plate moves axially in the barrel, it will interact with the barrel in a direction perpendicular to the motion of the flyer plate to consume kinetic energy. It can be seen from Figure 9 that at monitoring point 1, the velocity fluctuation perpendicular to the direction of motion of the flyer plate is no more than 30 m/s, which can be ignored compared to the acceptable range.

**Figure 10:**
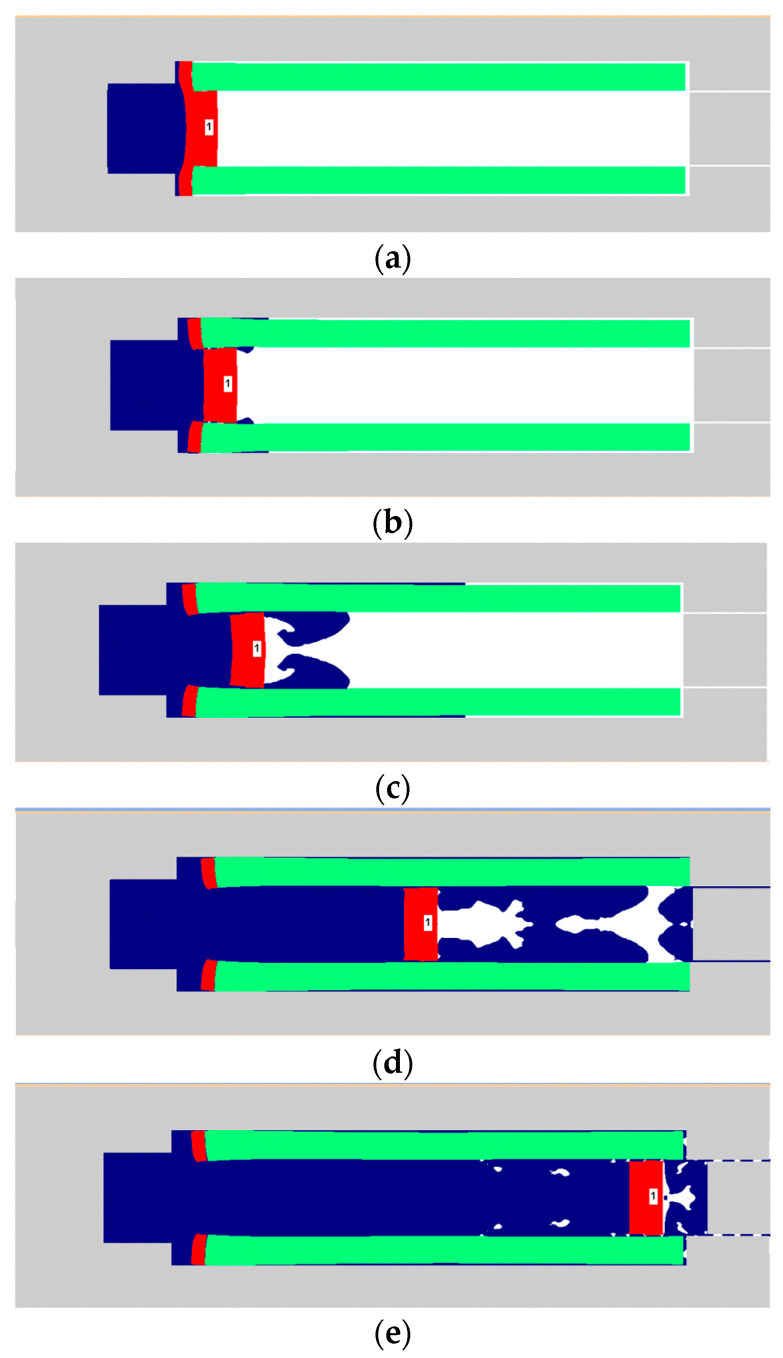
Figures of flyer plate motion process. (**a**) 15 μs, 0.3 mm. (**b**) 17 μs, 1.2 mm. (**c**) 20 μs, 3 mm. (**d**) 30 μs, 10 mm. (**e**) 46 μs, 20 mm.

The movement process of the flyer plate in the barrel is shown in Figure 10. At 15 μs, the peak pressure generated by the RDX deflagration reached the extreme value, and the flyer plate began to deform driven by the RDX deflagration. At 17 μs, the flyer plate has been completely sheared. Some of the RDX deflagration products propagate along the shear gap to the front of the flyer plate. The peak pressure of the RDX deflagration product has dropped sharply, and the flyer plate has moved 1.2 mm in the barrel at this time. At 20 μs, the flyer plate has moved 3 mm in the barrel, and the speed has reached 600 m/s. At 30 μs, the flyer plate has moved 10 mm, and the peak pressure in front of the flyer plate exceeds that at the rear for the first time. The deflagration products of RDX have diffused to the whole area of the barrel along the assembly gap of 0.1 mm. However, due to the small pressure and limited diffusion, the change of flyer plate velocity is not obvious. At this point, the speed of the flyer plate has reached a maximum value of 757 m/s. After 30 μs, as the pressure in front of the flyer plate is greater than that at the rear, the velocity of the flyer plate has dropped slowly from the extreme value of 757 m/s to 501 m/s.

### 3.2. Analysis of the Influence of Flyer Plate Mass on Its Motion Process

**Figure 11:**
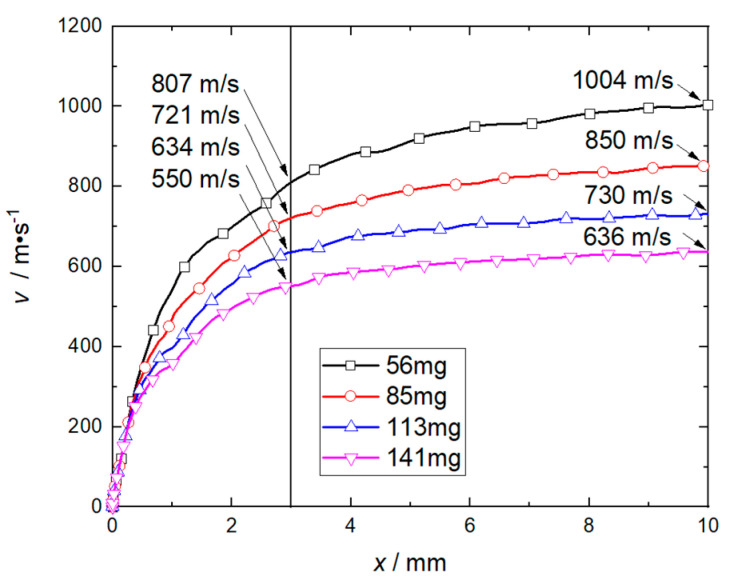
Velocity history under different flyer plate mass.

By changing the thickness of the flyer plate, the velocity histories of the flyer plates with different masses (56 mg, 85 mg, 113 mg, and 141 mg, corresponding to the total thickness of the flyer plate, respectively, 1 mm, 1.5 mm, 2 mm, and 2.5 mm) driven by the RDX charge of 75 mg were calculated. The deflagration products of RDX will affect the flow field in the barrel in front of the flyer plate after it has moved 10 mm, and decelerate it. Therefore, only the movement process of the first 10 mm is studied. Figure 11 shows the velocity histories of flyer plates with different masses. When the flyer plate moved 3 mm, its speed increased by 84 m/s, 87 m/s, and 86 m/s with every 28 mg reduction in its mass. When the flyer plate has moved 10 mm, the speed will increase by 154 m/s, 120 m/s, and 94 m/s with every decrease of 28 mg in the flyer plate mass. The calculation results show that when the length of the accelerating chamber is 3 mm, the speed of the flyer plate decreases linearly as its mass increases. When the length of the accelerating chamber is 10 mm, increasing the mass of the flyer plate has a significant inhibitory effect on its velocity, and this tends to be weakened.

**Figure 12:**
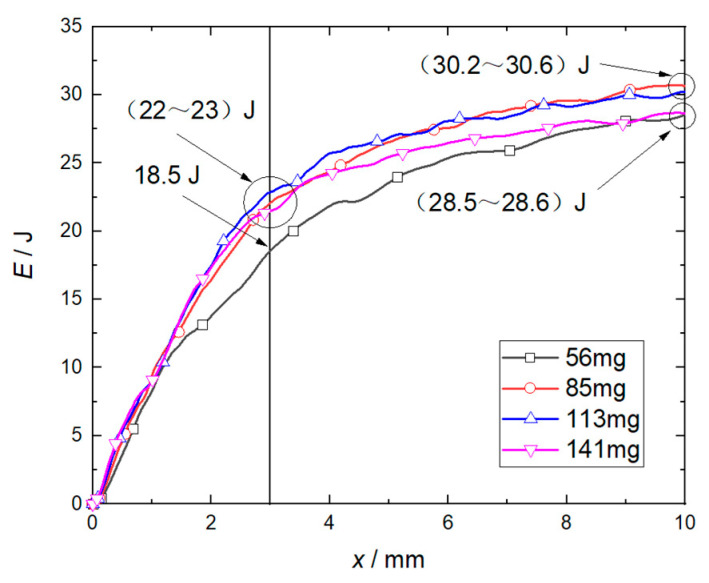
Kinetic energy history under different flyer plate mass.

Figure 12 shows the effect law of the flyer plate mass on its kinetic energy. The calculation results show that when the length of the accelerating chamber is 3 mm and the mass of the flyer plate is no more than 85 mg (the total flyer plate thickness of 1.5 mm), the increase of the mass of the flyer plate has a significant impact on its kinetic energy. When the mass of the flyer plate is ≥85 mg, the increase of the mass of the flyer plate has no obvious influence on its kinetic energy. When the length of the accelerating chamber is 10 mm and the mass of the flyer plate is ≤85 mg, the increase of the mass of the flyer plate has a significant effect on its kinetic energy. When the mass of the flyer plate is 85–113 mg, the increase of the flyer plate mass has no obvious effect on the kinetic energy of the flyer plate. When the mass of the flyer plate exceeds 113 mg, the increase of the mass of the flyer plate will inhibit the increase of its kinetic energy.

**Figure 13:**
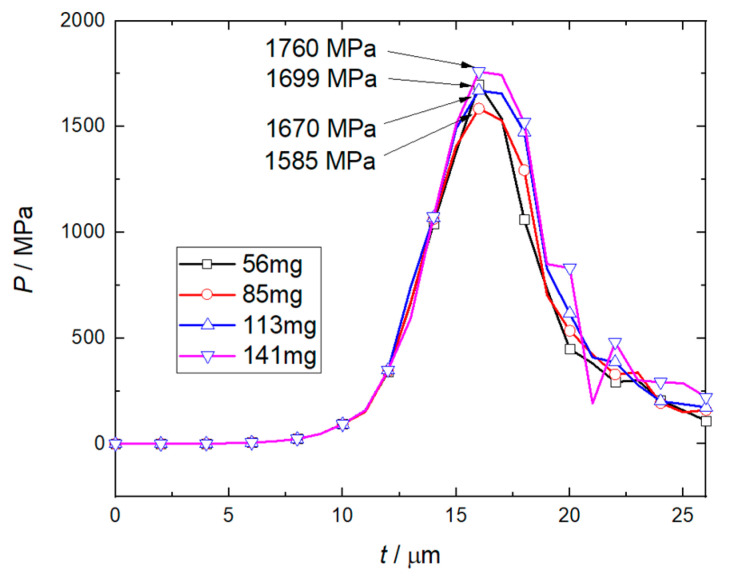
Effect of flyer mass on peak deflagration pressure of RDX explosive.

Figure 13 shows the effect of flyer plate mass on the peak pressure of RDX. The calculation results show that the increase of the mass of the flyer plate has a limited influence on the peak pressure of RDX, and the maximum value does not exceed 1760 MPa. This is because the main factor that promotes the growth of RDX peak pressure is the shear thickness of the flyer plate rather than the mass of the flyer plate that is moving in the accelerated chamber after being sheared.

**Figure 14:**
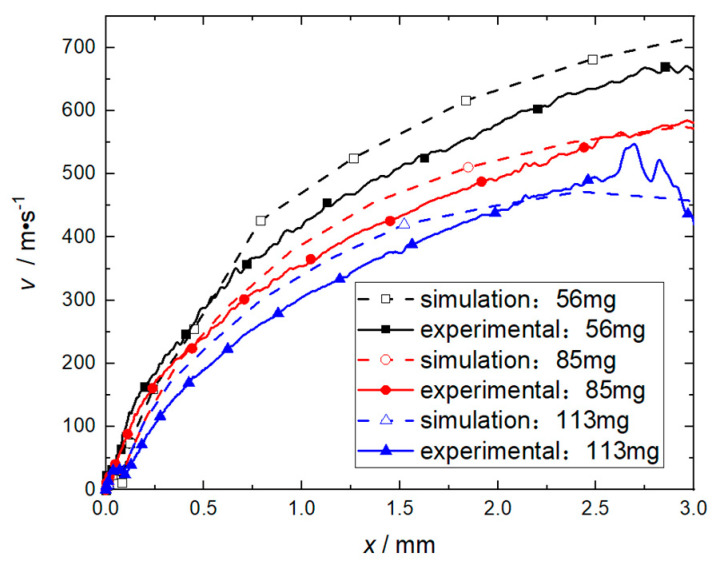
Effect of flyer plate mass on its velocity.

In order to confirm the effectiveness of the flyer plate mass of 85 mg (corresponding to a total thickness of 1.5 mm), when the length of the accelerating chamber is 3 mm, experiments with flyer plate masses of 56 mg, 85 mg, and 113 mg were conducted. The velocity histories of the flyer plate obtained are shown in Figure 14. The results show that the relative error of the numerical simulation relative to the experimental data under the 90% confidence level is $\left\{Y_{i}\right\}$ ≤ 45.2 m/s, and the velocity error at 3 mm is ≤6.7%.

**Figure 15:**
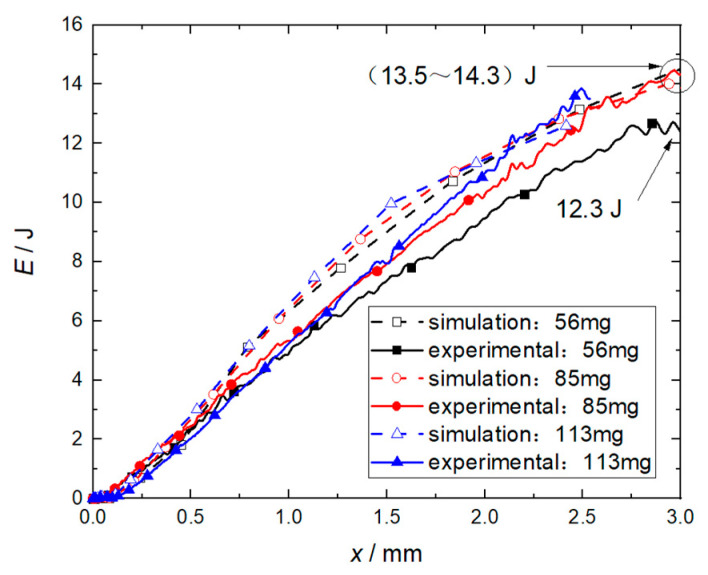
Effect of flyer plate mass on its kinetic energy.

It can be seen in Figure 15 that the impact of flyer plate mass on the kinetic energy of the flyer plate is small when the mass exceeds 85 mg, which is the same as that shown in Figure 11. Since the length of the accelerator chamber is reduced from 21 mm to 3 mm, the deflagration products and air of the RDX in front of the flyer plate are compressed in advance. This process has an inhibitory effect on the growth of the speed and energy of the flyer plate, resulting in a significant decrease in speed and energy. However, the corresponding numerical simulations and experimental results are in good agreement. Therefore, the critical mass value of the flyer plate under this structure can be set at 85 mg, and the corresponding total thickness is 1.5 mm.

The optimal mass of a flyer plate is the minimum mass at which the final kinetic energy of the plate is maximized, given the same amount of charge at the first level. The results of this study indicate that as the mass of the flyer plate increases, the plate’s velocity decreases and the peak velocity increment decreases, while the increase in kinetic energy transferred to the plate is not significant. When the charge weight of RDX is 75 mg and the length of the acceleration chamber is 3 mm, the kinetic energy increment obtained by the flyer plate is not significant when the plate mass exceeds 85 mg, hence the optimal mass of the flyer plate is 85 mg.

### 3.3. The Influence of the Mass of the First-Stage Charge on the Movement Process of the Flyer Plate

**Figure 16:**
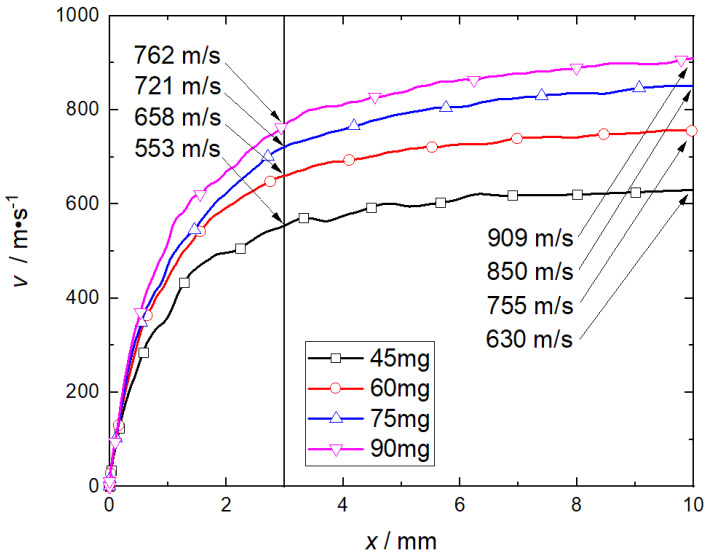
Effectof RDX explosive charge on velocity of flyer.

By changing the length of the charge, the velocity history of the flyer plate with a driving mass of 85 mg (corresponding to a total thickness of the flyer plate of 1.5 mm) was calculated with different RDX charge masses (45 mg, 60 mg, 75 mg, and 90 mg). Figure 16 shows the velocity histories of the flyer plate under different RDX charges. When the flyer plate moves 3 mm, the speed of the flyer plate increases by 105 m/s, 63 m/s, and 41 m/s with every 15 mg of the charge mass. When the flyer plate moves 10 mm, the speed of the flyer plate increases by 125 m/s, 100 m/s, and 59 m/s with every 15 mg increase of the charge mass. The calculation results show that when the charge mass of RDX exceeds 60 mg, the effect of increasing the charge mass on the speed of the flyer plate is limited.

**Figure 17:**
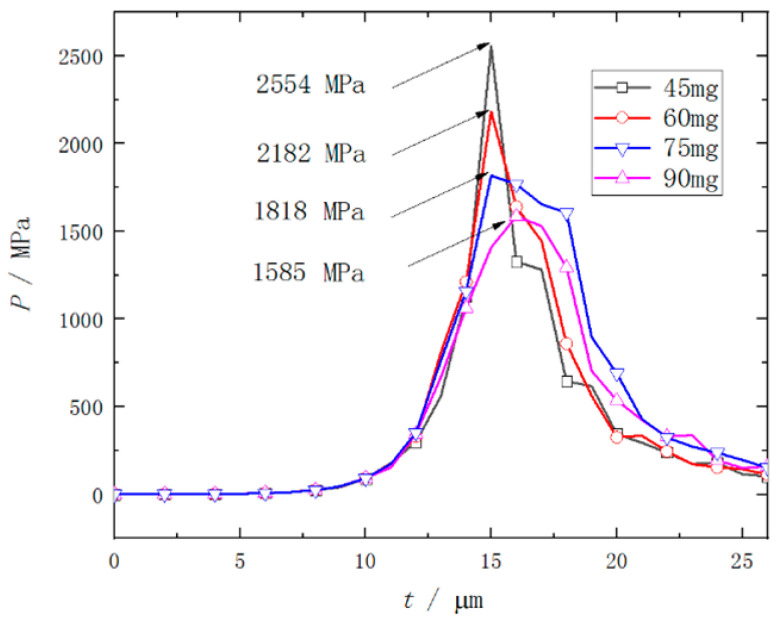
Effect of RDX explosive charge on its peak deflagration pressure.

Figure 17 shows the effect of different RDX charge mass on the deflagration peak pressure. The calculation results show that the peak deflagration pressure of RDX decreases by 372 MPa, 364 MPa, and 233 MPa with every 15 mg increase in charge mass. The shear thickness of the flyer plates is 0.6 mm, which means that the ability of the flyer plates to withstand the impact of the RDX deflagration is the same. Therefore, the stamping and shearing of the flyer plate began at about 15 μs, resulting in the deflagration pressure of RDX failing to grow to the maximum in a relatively closed cavity.

**Figure 18:**
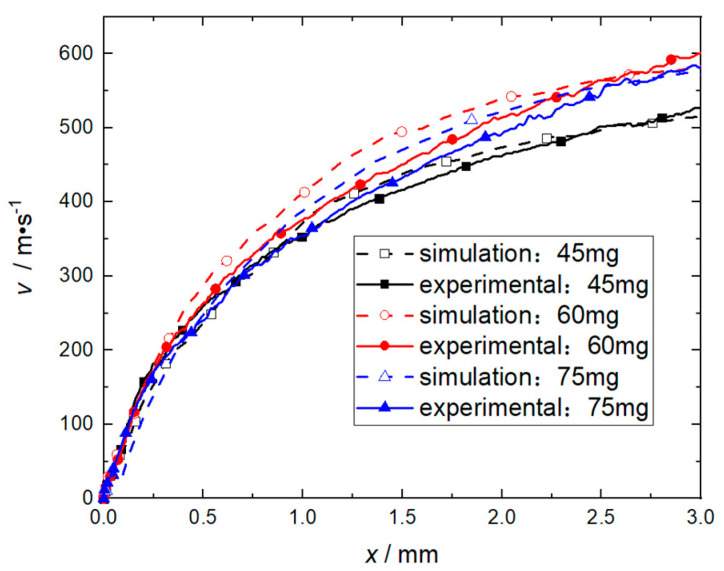
Effect of RDX explosive charge on velocity of flyer plate.

In order to confirm the effectiveness of the charge mass of 60 mg, when the length of the accelerating chamber is 3 mm, experiments with charge mass of 45 mg, 60 mg, and 75 mg were conducted, and the velocity history of the flyer plate is shown in Figure 18. The results show that at the 90% confidence level, the relative error of the numerical simulation relative to the experimental data is ≤57.8 m/s, and the velocity error $\left\{Y_{i}\right\}$ at 3 mm is no more than 5.1%. As the length of the barrel is reduced from 21 mm to 3 mm, the RDX deflagration products and air in front of the flyer plate are compressed in advance, which inhibits the increase in the speed and energy of the flyer plate. This process leads to a significant reduction in speed and energy; hence the corresponding numerical simulations and experimental results are in good agreement. Therefore, the critical value of the charge mass under this structure can be set at 60 mg.

The optimal first-level charge weight is the minimum weight at which the final kinetic energy of the flyer plate is maximized, given the same flyer plate mass. The results of this study indicate that as the RDX charge weight increases, the velocity of the flyer plate increases, while the peak velocity increment decreases significantly and the increase in flyer plate shearing time is not significant, but the corresponding RDX peak pressure decreases significantly. When the flyer plate mass is 85 mg and the length of the acceleration chamber is 3 mm, the kinetic energy increment obtained by the flyer plate is not significant when the RDX charge weight exceeds 60 mg, which is more favorable for the shearing of the flyer plate, hence the optimal RDX charge weight is determined to be 60 mg.

### 3.4. The Influence of the Length of the Accelerating Chamber on the Speed of the Flyer Plate

**Figure 19:**
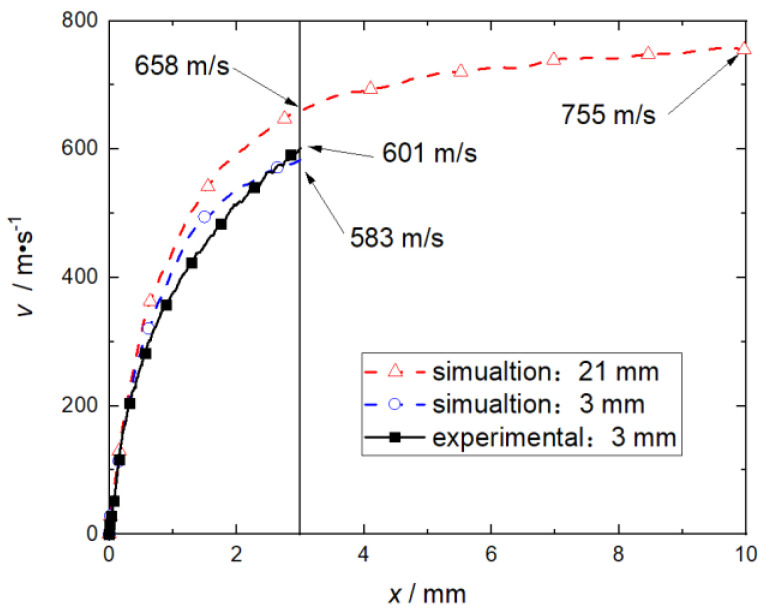
The effect of accelerating chamber length on flyer velocity.

In addition to the mass of the RDX and the flyer plate, the length of the barrel will also have a significant impact on the speed of the flyer plate. Therefore, under the optimal condition that RDX mass is 60 mg and flyer plate mass is 85 mg, the velocity variation of the flyer plate was studied, where the lengths of the accelerating chambers were 3 mm and 21 mm, respectively, as shown in Figure 19.

Figure 19 shows that when the length of the accelerating chamber increases, the speed of the flyer plate will increase accordingly. At the front of the flyer plate movement direction, the RDX deflagration products and air are compressed to inhibit the flyer plate velocity. Under the condition that the flyer plate moves 3 mm and the length of the barrel is 3 mm and 21 mm, the errors of the simulated flyer plate speed relative to the experimental data are 18 m/s and 57 m/s, respectively, which is within the acceptable accuracy range. After the flyer plate moves 5 mm, the speed change is not obvious. Considering the impact of RDX deflagration products on the flow field in the front direction of the flyer plate, the length of the accelerating chamber should not exceed 5 mm in practical engineering applications under this structure.

## 4. Conclusions

The Powder Burn deflagration model verified by the experiment can reproduce experimental data well under the 90% confidence level, and can be used for numerical simulation and pre-research of detonation propulsion under different types of charges with similar topological structures. Compared with the experimental results, the speed error of the flyer plate in the numerical simulation is ≤57.8 m/s, and the speed error of the flyer plate at the exit of the accelerating chamber is ≤6.7%. Under a given structure, the greater the charge mass of RDX, the greater the velocity of the flyer plate, and the charge mass has a significant impact on the peak deflagration pressure of RDX. The greater the mass of the flyer plate, the lower its speed. When the mass of the flyer plate is ≥85 mg, the increase of the mass of the flyer plate has no obvious impact on its kinetic energy. When the shear thickness of the flyer plate is the same, the total thickness has little effect on the peak deflagration pressure of RDX. During the movement of the flyer plate, the RDX deflagration products enter the flow field in front of the flyer plate and compress the air. Therefore, the length of the accelerating chamber should be within 3–5 mm to reduce the large disturbance of the RDX deflagration products on the flow field in front of the flyer plate and the inhibition of the growth of the flyer plate velocity. In the critical state (RDX charge is 60 mg, flyer plate mass is 85 mg, barrel length is 3 mm), RDX deflagration peak pressure is 2182 MPa, the flyer plate exits the barrel at a speed of 583 m/s, and kinetic energy is 13.5 J.

## Figures and Tables

**Table 1:** Main parameters for Powder Burn model of RDX explosive.

Parameter	Value
reference density	1.6 g/cm^3^
growth parameter G	300 mm^−1^
*C*_1_	1500 m/s
*C*_2_	0
r	0.01 mm

**Table 2:** Parameters for Mie–Gruneisen EOS of titanium alloy flyer [27].

Parameter	Titanium Alloy	Stainless Steel
Gruneisen coefficient	1.23	2.17
Parameter *C*_0_	5130 m/s	4569 m/s
Parameter S	1.028	1.49
Reference density	4.51 g/cm^3^	7.9 g/cm^3^

**Table 3:** Parameters for Johnson–Cook strength model and failure model of Titanium alloy [19,27,28].

Parameter	Titanium Alloy	Stainless Steel
Shear modulus	4.5 × 10^6^ kPa	7.2 × 10^7^ kPa
Yield stress	250 × 10^3^ kPa	510 × 10^3^ kPa
Hardening constant	1135 × 10^3^ kPa	792 × 10^3^ kPa
Hardening exponent	0.20	0.26
Strain rate constant	0.032	0.014
Thermal softening exponent	1.1	1.03
Melting temperature	1878 K	1793 K
Erosion strain	1.2	1.5
